# Health system response and recovery in Ukraine after 3 years of war: perspectives of front-line communities and internally displaced persons

**DOI:** 10.1093/heapol/czag049

**Published:** 2026-04-14

**Authors:** Mark Hellowell, Kateryna Kalendruz, Tetiana Bondar, Oleksiy Ganyukov, Jarno Habicht

**Affiliations:** Global Health Policy Unit, Social Policy, School of Social and Political Science, University of Edinburgh, 15a George Square, Edinburgh EH8 9LD, United Kingdom; World Health Organization Country Office for Ukraine, 9B, Hrushevskoho Street BC Summit, 3 floor. Kyiv 01021, Ukraine; Ukrainian Institute for Social Research after O. Yaremenko, 26, Panasa Mirnogo street, office 210, Kyiv 01011, Ukraine; Ukrainian Institute for Social Research after O. Yaremenko, 26, Panasa Mirnogo street, office 210, Kyiv 01011, Ukraine; World Health Organization Country Office for Ukraine, 9B, Hrushevskoho Street BC Summit, 3 floor. Kyiv 01021, Ukraine

**Keywords:** health systems, delivery of health care, health services accessibility, community participation, conflict, armed, warfare and armed conflicts, Ukraine, recovery of function

## Abstract

Communities affected by war can play a vital role in sustaining and restoring health services, yet their perspectives are under-represented in the evidence base. This study examines the experiences of patients and health personnel in three front-line areas of Ukraine, as well as persons internally displaced due to the full-scale invasion of February 2022. Our findings reveal community perspectives on the effects of war-related insecurity, workforce shortages, and infrastructure damage on health system functions and service availability. Respondents identified health worker shortages—driven by safety risks and resource scarcity—as among the most pressing concerns. Amid these challenges, however, recent health financing reforms, notably the Programme of Medical Guarantees and Affordable Medicines Programme, were often viewed as mitigating factors. Internally displaced persons (IDPs) generally reported positive care-seeking experiences in their host communities, though administrative and information-related barriers continue to limit access for some. Capacity constraints in areas hosting large numbers of IDPs are seen as placing further stress on service availability. Respondents valued the coordinated efforts of humanitarian actors and local authorities in restoring services, helping to maintain trust and social cohesion. Many were sceptical about the feasibility of large-scale reconstruction in the short term, prioritizing instead urgent security measures to protect facilities. However, they also acknowledged the importance of defining a long-term recovery strategy and identified workforce strengthening, integration of humanitarian services, and sustained financial protection as critical priorities.

Key messages

- After several years of full-scale war in Ukraine, communities in front-line regions describe severe disruption to health system functioning, but many respondents perceive health financing reforms as having helped to preserve financial protection and reduce access barriers, despite persistent challenges (e.g. insecurity, workforce shortages and informal payments).
- Internally displaced persons (IDPs) (specifically, those displaced following the 24 February 2022 invasion) generally report positive experiences of care in host communities, though some barriers remain—particularly in areas with restricted primary care capacity or where administrative and information-related hurdles limit patients’ enrolment with family doctors.
- Trust and social cohesion are critical enablers of recovery: hence, reducing informal payments, improving clarity on entitlements, and incorporating community/IDP feedback in recovery efforts can strengthen legitimacy and support sustained access.
- Respondents emphasize the importance of both short-term emergency responses and a long-term strategic recovery approach and identify workforce strengthening, integration of humanitarian services, and financial protection as critical priorities.

## Introduction

How health systems withstand, adapt to, and recover from shocks, including those related to conflict, has become a central focus of research (Kruk et al. 2015, Blanchet et al. 2017, Witter et al. 2023). Resilience depends on system-level factors such as effective leadership, adequate financing, and service delivery. In addition, the role of affected communities is emphasized in key strategic frameworks guiding emergency response and recovery. Grand Bargain 2.0—a set of commitments by donors and aid organizations—advocates for stronger localization of humanitarian action to shift power, resources, and decision-making closer to communities (IASC 2016, WHO Regional Office for Europe ‎2022). Community engagement is also a core principle of the Humanitarian–Development Nexus approach, helping to align short-term humanitarian and longer-term system interventions (Talisuna et al. 2023).

In this paper, we use ‘recovery’ to refer to the rebuilding, restoration, and strengthening of core health system functions and capacities in the context of war. Recovery in this sense extends beyond the mere reinstatement of pre-crisis service levels. It includes efforts to restore infrastructure and utilities, re-establish service delivery pathways, rebuild and support the health workforce, and strengthen governance and financing arrangements to improve resilience and equity (WHO Regional Office for Europe 2022, WHO 2024a).

In Ukraine, several years of war since Russia’s full-scale invasion on 24 February 2022 have placed extraordinary strain on the health system—destroying infrastructure, displacing populations, and disrupting access to care (Habicht et al. 2024). Overall, the costs of recovery in the health sector are estimated at US $19.4 billion for the 10 years to 2035 (World Bank et al. 2024). The government of Ukraine has defined a 5-year *Health System Strategy* (Cabinet of Ministers of Ukraine 2025). This frames recovery as a process extending beyond the restoration of pre-war functionality, adopting a ‘building back better’ approach aimed at enhancing efficiency, equity, and resilience to future shocks. Given Ukraine’s highly decentralized governance, community engagement has played, and will continue to play, a key role in the *Strategy’s* implementation (Arends et al. 2023, Rabinovych et al. 2023, Dale et al. 2024).

Previous research has documented the impacts of the war in Ukraine on health needs (Patel and Erickson 2022, Groot et al. 2024, WHO Regional Office for Europe 2025a, 2025b) and system functionality (Haque et al. 2024, Kim et al. 2024, WHO Regional Office for Europe 2024a), as well as the mitigating effects of health financing reforms undertaken from 2017 (Dale et al. 2024, Habicht et al. 2024).

Evidence shows that the war’s health system impacts have been greatest in the regions (oblasts) closest to the front line of armed conflict, in which the depletion of local resources has been most severe (WHO 2024b). Survey data show that populations in these areas seek care less often than elsewhere in the country and face greater barriers to essential services and medicines (WHO Regional Office for Europe 2024b, 2025a, 2025b).

Research has also documented the profound effects of mass displacement due to the war. In 2024, an estimated 3.6 million people were internally displaced—a four-fold increase compared to pre-invasion figure—some 2.2 million of whom required humanitarian assistance (OCHA 2024). This has led to a distribution of health needs across the country, placing additional strain on local health systems to meet the combined demands of host communities and internally displaced persons (IDPs) (WHO 2024b). While IDPs are also patients and care-seekers, displacement introduces additional administrative and informational hurdles (e.g. enrolment and documentation) and creates host-system capacity pressures that warrant focused analysis. For example, recent survey data indicate that IDPs are more likely than other Ukrainians to be without a registered family doctor—a pre-requisite for obtaining state-financed primary care and referrals to specialist and hospital care under the PMG (WHO Regional Office for Europe 2025b).

Qualitative research capturing the perspectives of war-affected communities in Ukraine remains scarce, however—reflecting a broader trend in the international literature, in which such perspectives are underrepresented (Durrance-Bagale et al. 2022, Truppa et al. 2024). This study addresses this gap by examining

- How residents of front-line oblasts and IDPs have experienced health system disruption and war-related barriers to care
- How health systems have adapted to sustain access to essential services
- What communities identify as priorities for both near-term recovery and longer-term reconstruction

The analysis is intended to inform Ukrainian national and subnational policymakers, managers of health facilities, and humanitarian and development partners involved in planning and financing health system responses and recovery efforts.

## Materials and methods

### Study setting

The study focuses on communities and local health systems in Dnipropetrovska, Khersonska, Mykolaivska, Poltavska, and Zaporizka oblasts. Since the Russian invasion of February 2022, Ukraine’s health system has sustained extensive physical and service disruption. As of 5 January 2026, the WHO’s *Surveillance System for Attacks on Health Care* had documented 2807 attacks on the system, resulting in 904 injuries and 225 deaths, with the use of heavy weaponry (artillery and mortar shelling, missiles, mines, and rockets) accounting for most of the damage (WHO 2025). Ukraine’s Ministry of Health estimated that, as of December 2024, nearly 2000 health facilities had been damaged, with 300 completely destroyed (Ministry of Health 2024).

Other evidence shows that the functionality of some facilities, particularly those located in areas that were temporarily under Russian military control in 2022 (and subsequently returned to Ukrainian administration), has been further compromised by removal of equipment due to looting, robbery, and theft (Haque et al. 2024). A WHO survey, published in October 2024, shows that the three southern front-line regions included in this study (Khersonska, Mykolaivska, and Zaporizka) have been among the worst affected (WHO 2024b), with the impacts on resources (primarily, buildings, and equipment), functionality, and accessibility especially severe in Khersonska (see Table 1).

**Table 1:** Status of health care resources, functionality, and accessibility in the three oblasts (WHO 2024b).

Impact	Khersonska	Mykolaivska	Zaporizka
Building damage			
Intact	43%	88%	87%
Partly damaged	48%	10%	10%
Fully damaged	9%	2%	3%
Equipment damage			
Intact	48%	89%	89%
Partly damaged	42%	9%	8%
Fully damaged	10%	2%	3%
Functionality			
Fully functioning	67%	95%	81%
Partly functioning	28%	3%	9%
Not functioning	5%	2%	10%
Accessibility			
Fully accessible	64%	96%	93%
Partly accessible	32%	4%	7%
Not accessible	4%	0%	0%

The war has also generated large population movements, both internal and external. By the end of 2024, several million IDPs were registered within Ukraine, with substantial flows from ‘front-line’ areas in the country’s south and east into more central regions (UNHCR 2025). Our study of IDPs focuses on regions identified by the International Organization for Migration as among the top 10 regions with the largest numbers of IDPs—Dnipropetrovska (509 000; 14% of national total), Mykolaivska (119 000; 3.3%), and Poltavska (168 000; 4.6%) (IOM 2024). Across the country, the war’s impacts on population health are indicated by increases in direct trauma and injury care needs, as well as increased mental health morbidity—for instance, 70% of respondents to a recent WHO survey reported symptoms of anxiety, depression, or severe stress in the year to April 2025 (WHO Regional Office for Europe 2025b).

In this context, ongoing reforms to health financing and service delivery—including the introduction of the ‘Programme of Medical Guarantees’ (PMG), including the ‘Affordable Medicines Programme’ (AMP), the establishment of a National Health Service of Ukraine (NHSU) as single state purchaser for this, and increased budgetary prioritization for primary care—may have helped to mitigate the war’s effects. Guaranteed entitlement to publicly financed health services based on (national) residency, central pooling, and strong purchasing is likely to have strengthened the capacity of local health systems to maintain core functions and sustain the availability of essential services, even in the most affected areas (Dale et al. 2024, Habicht et al. 2024).

### Data collection

The research was conducted between June and December 2024. Semi-structured qualitative interviews and focus group discussions (FGDs) were used to explore the perspectives of (i) care-seekers that are long-standing residents in one of three front-line regions of Ukraine (Khersonska, Mykolaivska, and Zaporizka oblasts), as well as health workers, facility managers, local health authority staff and local civil society representatives, and (ii) IDPs resident in one of three regions experiencing a large influx of population since the 2022 invasion (Dnipropetrovska, Mykolaivska, and Poltavska oblasts). We examine front-line residents’ experiences primarily in relation to disruption of local health system functioning under conditions of war, whereas we examine IDPs (who are also care-seekers) primarily in relation to continuity of access after displacement and interaction with host-area health systems.

The study was informed by literature on health system resilience and recovery in protracted crises (Kruk et al. 2015, Blanchet et al. 2017, Witter et al. 2023), with a particular focus on access to care and service delivery under conditions of insecurity and displacement (Cantor et al. 2021, Truppa et al. 2024). Topic guides for the semi-structured interviews and FGDs were accordingly designed to examine perceptions of disruption to service availability and quality (including infrastructure, utilities, supplies and workforce); care-seeking pathways and barriers, including those arising in displacement/host-area contexts (e.g. referral requirements, affordability, and informal payments); response strategies and the role of humanitarian support; and priorities for short- and long-term recovery and reconstruction (see Supplementary material). These domains were mapped to the three results objectives (front-line impacts; IDP access in host areas; recovery priorities).

Interviews were conducted with 34 selected key informants (subnational government officials, facility managers, and senior clinicians working in the front-line oblasts; see Supplementary material). Of these, 14 were held in-person in May 2024 and 20 were held online in September–November 2024. Interview guides were developed based on the objectives of the study and an analysis of existing literature (academic and grey) on health system responses and recovery efforts in Ukraine. Each interview lasted for 60–90 minutes and was audio recorded (with the agreement of each participant). Subsequently, audio recordings were uploaded to a secure server, transcribed (with all identifying information removed), coded according to a pre-defined matrix (see below), and translated into English for further analysis. The audio files were then deleted.

In addition, 14 online FGDs were conducted with a total of 114 participants (see Supplementary material) from three different groups, as follows:

**Group 1.** Care-seekers that are long-standing residents in one of the southern front-line regions. This group comprised individuals who had sought care for themselves or a household member within 6 months prior to the study and lived within 50 km of the front line of active hostilities in one of the three included oblasts (number of FGDs held = 8).
**Group 2.** Health workers employed in facilities located in one of the southern front-line regions. This group comprised health professionals, management, and/or support personnel employed in a facility within 50 km of the front line of active hostilities in one of three included oblasts. In addition, a small number of local civil society organization (CSO)/non-governmental organization staff located in these areas also participated in the FGDs (number of FGDs held = 3).
**Group 3.** People who had left their places of habitual residence due to hostilities after 24 February 2022 and had sought care for themselves or a household member in their host community in the last 6 months. One FGD meeting was conducted with IDPs living in Mykolaivska and one each with IDPs in two oblasts that are further from the front-line and host large numbers of IDPs (Dnipropetrovska and Poltavska) (total number of FGDs held = 3).

For Groups 1 and 3, a quota sampling design was adopted to ensure diversity in terms of age, gender, place of residence (urban/rural), and type of care sought (primary, secondary, specialized; inpatient, outpatient). For Group 2, the sampling was designed to ensure diversity in terms of professional category and level of institution.

Topic guides for the FGDs were developed in accordance with the study’s objectives and an analysis of published literature (academic and grey) on health system responses and recovery in Ukraine. Guides were reviewed and revised after piloting (this led, e.g. to the guides providing additional clarity on meanings of ‘recovery’). Each FGD lasted 90–120 minutes and was audio recorded with the agreement of all participants. Recordings were uploaded to a secure server, transcribed (with all identifying information removed), coded according to the pre-defined matrix, and translated into English. Audio files were then deleted.

Recruitment to the in-person key informant interviews was conducted by WHO country office staff, who recruited key informants for in-person interviews based on their knowledge and connections in each location. Before each interview, participants were provided with an information sheet. After reading this, each participant signed a written consent form.

For both the online interviews and the FGDs, the authors contacted local community partners with whom they regularly work to provide them with details of the study’s objectives, inclusion criteria, and research tools. Potential participants were identified in consultation with these partners and subsequently contacted by email. Before the online interviews and the FGDs, participants were provided with information about the study; after which, participants gave oral consent for their involvement in the research.

Ethical approval for the study was obtained from the WHO Ethical Review Committee (reference number: ERC.0004125) and a local ethical committee formed by the Sociological Association of Ukraine (reference number: no. 26/07-01). In relation to reflexivity, the multidisciplinary team combined Ukrainian researchers, WHO Ukraine Country Office staff, and an academic with long-standing Ukraine experience; interpretations were discussed across the author group (see full reflexivity statement).

### Data analysis

For the interview and FGD data, two of the authors developed an initial coding scheme informed by the study’s objectives and by existing literature on health system responses and recovery efforts in Ukraine, including both broader literature on health system resilience and recovery in conflict-affected settings and Ukraine-specific literature on health system responses, financing reforms, and recovery efforts (Kruk et al. 2015, Blanchet et al. 2017, Witter et al. 2023, Habicht et al. 2024, WHO Regional Office for Europe 2024b, 2025a, 2025b). Consistent with this, the coding matrix specified *a priori* categories corresponding to the three objectives (front-line impacts, IDP access in host areas, and recovery priorities), while additional codes and sub-themes were generated inductively through iterative reading and comparison of participants’ accounts. This scheme was discussed with the wider research team and refined accordingly. All transcripts were initially analysed in Ukrainian language, coded in Microsoft Excel by two of the authors, and cross-checked by the rest of the research team, after which the coding matrix was confirmed. Further analysis took place on the translated (English language) transcripts. Analysis of the qualitative data included triangulation with existing academic and grey literature, relevant aspects of which are, where necessary, presented in the results section alongside the primary data.

The final interpretation of data was discussed among and agreed by all of the authors.

## Results

The results are organized into three main sections:

- Perspectives of communities in the front-line areas (care-seekers, health workers, local authority staff) on the war’s health system impacts (3.1)
- IDP care-seekers’ perspectives on service availability and access in host areas (3.2)
- Across both of the above groups, the views, expectations, and aspirations for early and long-term health system recovery (3.3)

These three sections reflect the study objectives; within each, we report sub-themes that emerged from participants’ accounts and illustrate them with verbatim quotations.

### Community perspectives on the war’s impacts on local health systems

#### Legacies of occupation and ongoing security threats

Several respondents described their experiences of occupation and highlighted its ongoing effects on local health systems. These accounts align with evidence that forms of ‘militarization’—that is, interference in civilian health assets by military forces—have been extensive in temporarily occupied areas of Ukraine and have included restrictions on access to services and medicines (e.g. through non-medical use of health facilities) (Kim et al. 2024). Respondents emphasized that these dynamics have had lasting implications for local facilities, particularly in relation to available equipment and health workforce capacity.

All hospitals lack equipment. When we were under occupation, everything was stolen, everything - yes, even the old ambulances. The orcs [Russian military personnel] took them. (Participant in Focus Group 4 (patients/carers), Mykolaivska)

Doctors left [the occupied areas] because the Russian military started attacking them or forced them to work for the occupying forces. And now that time has passed, people have become established in their new locations; they have built a new life there. (Chief nurse, Khersonska)

Across the front-line regions, several health worker respondents cited the impacts of the war’s proximity—including frequent shelling, missile strikes, and targeted attacks on hospitals and ambulances—as major drivers of health workers’ decisions to re-locate.

Everyone has their own motives [for leaving] - some have a family they want to keep safe, and many people have experienced trauma. There was a doctor who worked for us; her apartment was hit by a rocket while she was on duty. Later, she was in tears. She said to me, ‘I can’t sleep, I can't work properly, I’m sorry’. What can I say? What can I say to her – ‘Don’t go’? (Senior doctor, Zaporizka)

In general, among our respondents, the shortage of health workers was perceived to be the primary driver of access barriers. In addition, in some areas further from the front-line (e.g. the regional capital city of Zaporizhzhia), respondents noted that the reduction in health workers has been accompanied by increased demand from IDPs and Ukrainian soldiers in need of care.

Approximately, in our area, we have more than 9000 registered IDPs, and that is a challenge for us. Each of our family doctors has to care for many more patients than we are capable of. Instead of providing care for, perhaps, 1500 people, my colleagues and I have up to 3000 to care for. That is the general situation in family medicine in this area. (Senior doctor, primary care clinic, Zaporizka)

In turn, workforce shortages are perceived to have placed heavier workloads on remaining health workers, contributing to rising levels of stress and burnout, with some health workers concerned this had led to a reduction in care quality:

At the beginning of the war, two family doctors were taken away to the war. That left two family doctors for 8000 people. It’s very hard, it’s a lot of work, and doctors don’t always manage to give people the attention they need. (Participant in Focus Group 12 (health workers), Mykolaivska)

#### The war’s impacts on geographical barriers

Continued military assaults on health care infrastructure exacerbate long-standing geographical barriers to access. Several respondents stated that, as health facilities continue to be targeted by Russian military forces, they are unsafe for patients, families, and staff. In addition, transport infrastructure is targeted, such that travelling to and from health facilities is perceived to be dangerous.

To get an emergency case out of [town X], you have to do it yourself. No ambulance will come. (Participant in Focus Group 1 (patients/carers), Zaporizka)

There is a problem to get to a medical facility because, in fact, the entire service area is under constant shelling. (Participant in Focus Group 14 (health workers), Khersonska)

In many areas close to the front line, the remaining population disproportionately comprises elderly and/or vulnerable people, often with complex health needs. Many need social support as well as health services. In such areas, adjustments to NHSU capitation payments, designed to recognize the additional costs of treating elderly patients, may be insufficient to meet average costs in contexts in which there is a high proportion of patients with complex needs.

The population of Kherson has decreased by almost three to four times. Mostly, unprotected groups of the population remain. These are elderly people, people with disabilities or socially vulnerable groups. (Subnational health authority official, Khersonska)

More generally, the fact that NHSU payments ‘follow the patient’ (e.g. in the form of capitation payments at primary care level) can give rise to financial challenges, restricting local supply of primary care.

Primary care suffers because funding depends on the number of declarations [i.e. enrolled patients], which are sharply declining, even now. It has a very big impact and it is difficult for primary care facilities to survive. (Participant in Focus Group 14 (health workers), Khersonska)

In locations where access to government/municipal health facilities is challenging, humanitarian agencies are playing a critical role (UNHCR 2025). In such contexts, barriers to access are being addressed by new forms of service delivery, including modular primary care facilities established by the WHO and other organizations in areas of unmet need, and mobile teams employed by humanitarian agencies. This fact was highly salient to, and welcomed, by many respondents.

I’m happy that every month, every three Sundays, a doctor comes to see us. It’s a therapist, a cardiologist, sometimes a gynaecologist. For example, my child had problems with his spine and back. I didn’t need to travel to [facilities in local urban centres]. They came to us, wrote me a prescription, told me what medication I needed, what it was. I am satisfied with this. (Participant in Focus Group 5 (patients/carers), Mykolaivska)

#### The war’s impacts on financial barriers

Since 2018, Ukraine has implemented reforms aimed at achieving universal population coverage through introducing a defined benefit package, the PMG, which includes the AMP. Centralized pooling of funds under the PMG has likely played a critical role in sustaining service coverage in front-line oblasts, many of which have experienced substantial losses in locally generated revenue (Habicht et al. 2024). Moreover, the universality of the system ensures that IDPs retain full legal entitlement to health services in their host communities, facilitating access to and continuity of care.

Among our respondents, patients/carers and health workers perceived the PMG and AMP as generally effective in reducing financial barriers.

I was an inpatient not so long ago, for 3 weeks in [X] hospital. I tell you, it has changed for the better. I didn’t pay anything to anyone. I was operated on, I was given all the therapy. I was fed. Well, I was surprised. For our hospital, this is new. (Participant in Focus Group 2 (patients/carers), Zaporizka)

The reform has had a lot of impact [on patients]. Take the example of asthma. Before the reform, people were not receiving proper care because combined inhalers were too expensive for them. Now that combined inhalers have been added to the [AMP], a lot of patients finally have a way to treat their symptoms, because they receive those inhalers for free. (Senior doctor at a primary care facility, Zaporizka)

However, the focus group and interview data also reveal a diversity of experiences concerning the financial conditions of access. In some accounts, barriers have arisen due to limitations in the depth of coverage in the PMG and AMP packages:

My husband was seriously wounded [during the occupation]. I was confused by the fact that while he was in hospital he was treated free of charge, and then when he was discharged, rehabilitation was also free of charge. But he was prescribed medicines for his injury, and they were quite expensive. He had to buy them at his own expense. (Participant in Focus Group 6 (patients/carers), Mykolaivska)

In contrast, in other accounts, the challenges appear to arise, not from formal limitations, but due to ‘opportunistic’ behaviours by some care providers—stimulated by the disruption and scarcity generated by the war-time conditions.

More than once I have faced a situation where I come to see a specialist and they prescribe some tests. And I ask, can I have these tests done free of charge at your hospital? The answer is yes, but you will have to wait a very, very long time and you’d better do it in [a private facility]. I understand [that the private facility] probably gives them some kind of incentive later on. (Participant in Focus Group 1 (patients/carers), Zaporizka)

### Internally displaced persons’ perspectives on health care availability and access in their host regions

As noted, the fact that all residents of Ukraine have, in principle, guaranteed financial coverage under the PMG and AMP—regardless of their location in the country—appears to have supported continuity of access to health care for IDPs. In the April 2025 WHO adult health needs survey, access patterns for IDPs were broadly similar to those of non-displaced people for some indicators (e.g. the report notes no significant difference in reported receipt of primary care: 5% vs 4%). However, IDPs reported specific barriers such as refusals to provide services/consultations (29% vs 12%) and security concerns when accessing chronic care (33% vs 10%) (WHO Regional Office for Europe 2025b), though confidence intervals/standard errors are not reported for all subgroup comparisons.

These findings are broadly consistent with those emerging from our FGDs with IDPs, which describe generally positive experiences of care-seeking after displacement. Several IDP respondents reported that they had not encountered discrimination in accessing care, and their legal entitlement to free care had been generally accepted by providers.

The doctors treated me normally, it didn’t matter whether I was an IDP or not. Everything was normal. (Participant in Focus Group 9 (IDPs), Mykolaivska)

When we moved to [Location X], we immediately went to the local family doctor. We went to the clinic, we went to the hospital, everything was free, there were no problems. Our child is recovering from a kidney operation, and we have been to the hospital for inpatient treatment, laboratory tests – all free. (Participant in Focus Group 11 (IDPs), Poltavska)

At the same time, participants described challenges in navigating administrative requirements and information gaps; health system capacity constraints in host communities; and, for some, uncertainty about what should be free under the PMG.

IDPs must sign a ‘declaration’ with a local family doctor to access prescriptions for AMP medicines and referrals for PMG services at local outpatient or inpatient facilities. However, a lack of clear information for IDPs may impede their ability to make a declaration and thus access care:

[There is a] lack of information. If, for us, for displaced people, there was someone who said, ‘Ah, you need to do this, register this, sign a contract with this doctor’, and so on, then it would be much easier [to know what to do]. I mean, to make it a bit easier for people. Because people are already scared. (Participant in Focus Group 9 (IDPs), Mykolaivska)

Capacity constraints in host communities could further limit effective coverage, even where entitlement was clear. Some IDP respondents highlighted that although access to a family doctor is formally guaranteed, care capacity in host regions with large numbers of IDPs is often overstretched.

With such a large influx of people into this area, there is no place for us to make a declaration. The doctors explain it by saying that they have accepted a certain number of people and they are unable to enrol any more. (Participant in Focus Group 10 (IDPs), Dnipropetrovska)

This problem can be exacerbated by PMG rules, under which capitation payments to family doctors begin to decrease once their patient panel exceeds 1800 (WHO Regional Office for Europe 2023), creating a disincentive for doctors to enrol patients once they have a panel of 1800 or more. This might impede IDPs’ access to primary care in some localities—and thus, also, to medicines and specialized care.

At the specialist care level, too, local health systems in areas experiencing a considerable influx of IDPs have found it challenging to address increased demand—resulting in long wait times for some services.

In hospitals, the queues are a nightmare. You go in and there is a crowd of people outside each office. (Participant in Focus Group 11 (IDPs), Poltavska oblast)

Finally, some IDPs reported a lack of access to free hospital care—with out-of-pocket payments being incurred in some cases—alongside a lack of transparency about which services are covered under the PMG and which are not.

While vulnerability to financial exploitation (e.g. informal payments, abuse of dual practice, undue referrals to private care settings) is not restricted to IDPs, they may be more vulnerable to such adverse behaviours—for example, if they lack a formal referral and/or are less familiar with the conditions of access care in local hospitals.

Furthermore, the financial consequences for IDP households are greater, on average, given higher levels of impoverishment and unemployment within this group (IOM 2024).

We had an MRI [magnetic resonance imaging] scan – we paid, we had a CT [computed tomography] scan – we paid, and wherever we went, whenever we needed some tests, some examinations, ultrasound, we paid. They say: ‘if you want it for free, go to Poltava [oblast capital], but bear in mind there is a queue for 3–4 months in advance.’ So, in the end, we had to pay. (Participant in Focus Group 11 (IDPs), Poltavska)

### Perspectives on health system recovery

Participants described recovery as a process already underway, but constrained by continuing insecurity and resource disruption. Participants in the health workers subgroup emphasized that effective recovery under war-time conditions depends on close coordination between emergency responses and health system structures. Among other respondents, the efforts of local recovery stakeholders—governments, humanitarian agencies, CSOs, and health workers—to quickly restore services amid ongoing attacks were recognized and valued and frequently linked to trust in decision-makers.

Participants’ accounts clustered around four linked themes: (i) early recovery efforts already underway; (ii) immediate wartime priorities focused on safety and continuity of essential utilities; (iii) workforce constraints as the central recovery challenge; and (iv) longer-term recovery and return.

#### Early recovery efforts already underway and valued by communities

The local authorities and voluntary organizations did a great job after what the Russians did to our villages and cities [during occupation of part of Mykolaivska oblast in 2022]. They provided everything they could. (Participant in Focus Group 6 (patients/carers), Mykolaivska)

There was no pharmacy, no delivery of medicines [during the occupation of Kherson city in 2022]. Our hospital was heavily damaged by the orcs [Russian military personnel] but it was rebuilt very quickly. When I came home two months after de-occupation, the hospital was already functioning. (Participant in Focus Group 7 (patients/carers), Khersonska)

Local government staff also recognized the efforts made by health workers and facility managers to sustain core functions in the challenging conditions of the war's early phase:

The city of Mykolaiv was at risk of being occupied [in the spring of 2022]. Pharmacies closed. Medicines were not delivered. But none of the health facilities closed, and of the senior managers of the facilities, only one left his job. The rest stayed and worked. There were times when we worked around the clock and did not return home for several days. (City official 2, Mykolaivska)

#### Immediate wartime priorities: protecting facilities and maintaining essential utilities

Restoring health facilities has been a central focus of recovery efforts. As of December 2024, Ukraine’s Ministry of Health reported that, of the 2235 facilities damaged or destroyed during the war, 576 had been fully restored and a further 372 partially rebuilt (Ministry of Health of Ukraine 2024). However, several respondents voiced scepticism about the practicality of large-scale rebuilding in the face of escalating attacks on civilian infrastructure (United Nations Security Council 2024) in the frontline areas.

Have you seen the attacks on our infrastructure? Talking about rebuilding now – it’s like trying to skin a live bear. (Senior doctor, Zaporizka)

Many respondents emphasized the urgent need for additional resources to strengthen the physical protection of health facilities, including provision of adequate shelters and measures to ensure uninterrupted access to basic utilities including water and energy.

The situation has worsened, because FPV [first-person view] drones are now hitting our facilities – also long-barrelled artillery, which reaches the centre of Kherson. They’ve built shelters, put sandbags near the windows, providing at least some chance for the hospitals that have survived to continue to do so. But we need stronger shelters or to rearrange basements so that they can accommodate all staff and patients. (Local CSO representative, Khersonska)

Some respondents observed that knowledge of how to address these priorities has evolved over time, creating an opportunity to gather and share evidence on the most effective approaches.

A missile attack on [a local multi-profile hospital] completely destroyed the outpatient building. A children’s hospital was hit by cluster bombs. And then, we had no water supply for a long time, for several months [after the destruction of the Kakhovka Dam]. We’ve had to build wells for almost every hospital. Then we had the blackouts. Our facilities had to be backed up by generators. And we had a few problematic issues with shelters. We had to remodel basements and build new structures. It's only now, actually, that we understand all the necessary components. (Subnational health authority official, Mykolaivska)

#### Workforce constraints as the central recovery challenge

Beyond ensuring basic safety for patients and staff and maintaining uninterrupted access to essential utilities like water and energy, respondents identified addressing workforce shortages as the most critical priority for recovery during war-time.

The material base is improving already – it’s clear that more equipment is coming. But I don't know about the doctors, how to lure them back – that's the key…. A piece of iron alone won’t cure anything, right? There has to be a person who knows how to use it. (Participant in Focus Group 8 (patients, carers), Khersonska)

Respondents suggested that additional financial and/or in-kind incentives are needed to support staff retention in war-affected regions—but funding for this is lacking.

The main challenge is the workforce. We try our best to keep people, we try to increase salaries, but our ability to do so is not endless. We might say, let’s increase salaries by 3000 hryvnia, but this is on the budget of the facility. In our region, budgets are very limited. (Senior hospital doctor, Zaporizka)

The same respondent also suggested that support for recovery of the workforce should focus on front-line areas, where need is most acute.

We need to bear in mind these differences [across regions]. You know, there may be a surplus of health workers in Zakarpattia [a region in the western part of Ukraine]. Lviv may have no problems with personnel. But who will we have here? (Senior doctor, Zaporizka)

#### Longer-term recovery, return, and the need for a clear strategic vision

Respondents from the IDP group emphasized that health system recovery in their location of origin will be crucial to their ability to return.

Eventually the hospitals will be rebuilt again – I think this will be the first step towards people returning. (Participant in Focus Group 9 (IDPs), Mykolaivska)

I am from the city of Bakhmut in the Donetsk region. Before the war, we had a great children's hospital, restored, restructured, with new equipment. We had a lot of our perinatal centres fully modernized, and we had a diagnostic centre. It is a great pity that we have lost all of this now…. My town was completely destroyed. We were scattered like peas all over Ukraine. There is not a single surviving building there. But after the war, we will repair all the destroyed and damaged buildings. We will rebuild it from scratch. (Participant in Focus Group 11 (IDPs), Poltavska)

Early recovery efforts—focused on restoring basic safety for patients and staff, and ensuring access to essential utilities—have helped build trust in health system decision-makers, including central and local governments, humanitarian and development actors, and other stakeholders. However, several respondents emphasized the need for a clear long-term vision—creating a basis for coordinated action, and a transition to longer-term recovery strategies—as pre-requisites for encouraging the return of IDPs and health workers to the most war-damaged areas.

Our long-term challenge [for recovery] is to create a strong medical ecosystem in this region with health facilities that are strong enough to allow young students to realize their potential so that they will have a desire to come to and work in the facilities in this region. (Subnational health authority official, Mykolaivska)

## Discussion

Previous research has documented thousands of attacks on Ukraine’s health system (WHO 2025), the displacement of millions of people (UNHCR 2025), and extensive disruptions to essential services (WHO Regional Office for Europe 2025a, 2025b), compounding long-standing socio-economic and geographic inequities in access and placed unprecedented strain on service delivery (Haque et al. 2024).

Our findings show how these systemic shocks are reflected in the lived experiences of people in the war-affected communities. Participants described barriers to care arising from active hostilities—including the risk of shelling when travelling to, or receiving care in, health facilities—as well as acute workforce shortages and, particularly for IDPs, administrative hurdles, and information gaps. In many accounts, coordinated action by local authorities and humanitarian actors was seen as important for sustaining trust in health system response and recovery efforts, aligning with evidence from other conflict-affected settings (Kruk et al. 2015, Blanchet et al. 2017, Jewett et al. 2021, Durrance-Bagale et al. 2022, Talisuna et al. 2023, Witter et al. 2023).

### Front-line areas: key barriers to care under active hostilities

In front-line areas, care-seekers, health workers, and local officials described how the legacies of occupation and continuing insecurity—including attacks on health facilities—shape barriers to care. Across the interviews and FGDs, workforce shortages emerged as the single most salient issue for respondents, echoing prior evidence that insecurity-related departures of health workers undermine health system functioning (e.g. Fouad et al. 2017). Participants pointed to localities very close to the front line, or otherwise subject to frequent attack, as facing the most severe barriers to care. In many such areas, sustaining a consistent local service presence is difficult (because staff leave and facilities are repeatedly disrupted), while travel to alternative facilities is unsafe—as roads and transport (including ambulances) are exposed to shelling. The consequences were perceived to fall most heavily on older and clinically vulnerable people who remain in these localities and require more frequent care and support.

### Financial guarantees: mitigating (but not eliminating) the war’s impacts on access

In alignment with previous research (Habicht et al. 2024, Shuftan et al. 2025), respondents’ accounts indicate that health financing reforms—particularly the PMG and AMP—were widely perceived as helping to preserve financial protection and entitlement to services amid war-related shocks. Several participants reported receiving, at no cost, forms of care for which they had expected to pay. However, participants in both the ‘settled’ and displaced groups described being asked to make informal payments in some cases, suggesting that formal entitlements are not always realized in practice. While informal payments are a long-standing feature of Ukraine’s health system, war-related disruption and scarcity may further enable such behaviours (Williams and Horodnic 2017).

### For internally displaced persons, entitlement in principle, barriers in practice

IDPs in our study generally reported positive experiences of care-seeking in their host communities, reinforcing the view that legal entitlement under the PMG/AMP has supported continuity of access. However, their accounts point to recurring barriers that are specific to (or intensified by) displacement. In particular, participants described difficulties navigating administrative requirements and information gaps relating to enrolment with a family doctor, as well as capacity constraints in host communities—especially where a large population inflow coincides with workforce depletion. These findings align with evidence that displaced populations tend to face additional access barriers compared with settled populations (Cantor et al. 2021). They also suggest that enrolment status (i.e. whether a declaration has been signed) is a key determinant of practical access to primary care, AMP medicines, and referrals to specialist care.

Participants’ accounts of constrained primary care capacity in some host areas are also consistent with the possibility that existing rules and incentives (including the tapering of capitation payments above a panel size of 1800) may unintentionally discourage additional enrolment in precisely those locations where needs have risen sharply (WHO Regional Office for Europe 2023). At specialist care level, too, IDPs described long waits and congestion in facilities serving some high-influx areas, suggesting that increased demand cannot always be met by available workforce capacity—again highlighting recruitment and retention as critical bottlenecks.

### Transitioning from ‘early’ recovery to long-term system strengthening

Across participant groups, recovery was described as a process already underway, but constrained by continuing insecurity and disruption. Participants valued coordinated efforts by local communities, health authorities, and humanitarian agencies in restoring health system functioning amid continuing attacks. This coordinated approach—resonating with key strategic principles of Grand Bargain 2.0 and the Humanitarian–Development Nexus—is perceived to have fostered trust in decision-making and built social cohesion, attributes that are likely to ease the transition to longer-term health system strengthening and the complex trade-offs involved (Witter et al. 2023).

At the same time, respondents expressed scepticism about such long-term recovery efforts during the acute phase of the war, prioritizing instead measures focused on immediate needs—in particular, protecting facilities and securing continuity of essential utilities such as power and water.

While infrastructure restoration was acknowledged as necessary, respondents often framed it as insufficient without staff, security, and basic enabling conditions. In particular, participants repeatedly identified workforce strengthening as the central requirement for sustaining services and enabling longer-term recovery. Several IDPs emphasized that visible progress in rebuilding health services in their places of origin would be important to any future return.

### Implications for response and recovery priorities

Taken together, participants’ accounts—alongside guidance and evidence on recovery and Humanitarian–Development Nexus approaches in conflict-affected settings (e.g. WHO Eastern Mediterranean Regional Office 2021, WHO Regional Office for Europe 2022, WHO 2024a)—suggest several priorities for future response and recovery efforts. These are differentiated by role, but are intended here to operate as a coherent whole.

At the national level, state authorities (including NHSU and MoH) can reinforce equitable access and financial protection by maintaining predictable financing for the PMG and AMP and by strengthening oversight and enforcement to reduce informal payments and improve transparency. National coordination is also required for recovery investments that protect facilities and secure continuity of utilities (power, water, connectivity), alongside workforce safety and retention measures. In addition, participants’ accounts of constrained primary care capacity in some host areas suggest that national purchasing rules and incentives may need review—particularly where capitation payment ‘tapering’ above certain panel sizes may unintentionally discourage additional enrolment in high-influx localities (WHO Regional Office for Europe 2023).

At the oblast and municipal levels, authorities and other health system leaders can act to translate national enabling conditions into functioning local care pathways for both settled and displaced populations. Such action should focus on: assessing needs and capacity, organizing and adapting local networks and referral routes (including transport and outreach where required), and improving communication so that people can understand entitlements and navigate services—including practical support to complete family doctor enrolment, which participants’ accounts suggest is a key gateway to accessing AMP medicines and PMG referrals (Cantor et al. 2021, WHO Regional Office for Europe 2022, WHO 2024a). This could include clearer signposting, simple step-by-step guidance, and locally accessible support points to help displaced people register and access referrals in practice. These actions align with participants’ accounts of administrative/information barriers and host-area capacity constraints as key barriers to access.

For NGOs and international partners, support should complement, and align with, national and subnational authorities’ efforts. This means, for example, providing flexible surge capacity where access is most constrained (including mobile primary care teams and visiting specialists in localities very close to the front line, or otherwise subject to frequent attack, where travel to facilities is unsafe and service availability is severely restricted), coordinating with state authorities (and with each other) to ensure capital investments target needs and align with government/oblast-defined service reconfiguration plans, and supporting participatory planning and feedback mechanisms so that community and IDP perspectives inform priority setting, implementation, and monitoring. Across these areas, partners should align these inputs with national and oblast strategies to avoid parallel pathways and support coordinated approaches to response and recovery (Jewett et al. 2021, WHO Eastern Mediterranean Regional Office 2021, Durrance-Bagale et al. 2022, Talisuna et al. 2023).

## Conclusion

After several years of full-scale war, communities in Ukraine’s front-line regions and IDPs in ‘high-influx’ parts of the country describe a health system that continues to function—but is under severe strain from insecurity, damaged infrastructure, disrupted utilities, and acute workforce shortages. Financial reforms centred on the PMG/AMP and NHSU purchasing tend to be viewed as making an important contribution to sustained access and financial protection, but challenges remain—for example, informal payments, and administrative and information-related barriers for IDPs. Priorities for the next phase of response and recovery include (i) sustaining predictable financing and strengthened oversight to safeguard equitable access; (ii) protecting facilities and ensuring continuity of essential utilities; and (iii) stabilizing and supporting the workforce, alongside better integration of humanitarian support with routine primary care and referral pathways. Systematic incorporation of community and IDP perspectives into recovery planning and monitoring can strengthen legitimacy and effectiveness. Doing so can also help maintain trust and social cohesion—which are in Ukraine, as in other settings, critical enablers of effective and sustainable recovery.

## Supplementary Material

czag049_Supplementary_Data

## Data Availability

Due to their sensitive nature, data from the interviews and focus group discussions, on which the main findings of this article are based, remain confidential.

## Reflexivity statement

The authors include two females and three males. Two of the authors (K.K. and J.H.) work at the WHO Country Office in Ukraine. Based in Kyiv, they have long-standing experience of working closely with the government of Ukraine and other stakeholders to address health system and humanitarian challenges in the country. One of the authors (M.H.) is a specialist in health systems strengthening, based outside of the country, but with direct, extensive, and ongoing experience of working in Ukraine. Two of the authors (T.B. and O.G.) work at the Ukrainian Institute for Social Research after O. Yaremenko—a non-governmental organization based in Kyiv. They have extensive experience in conducting qualitative fieldwork in Ukraine.
