# Supplementary material for: Health system response and recovery in Ukraine after 3 years of war: perspectives of front-line communities and internally displaced persons
Source: Health Policy Plan. 2026 Apr 14;41(5):719–30. doi: 10.1093/heapol/czag049 (PMC13187625; doi:10.1093/heapol/czag049)
Supplement: czag049_Supplementary_Data [file czag049_supplementary_data.docx]

# Supplementary File

## Part A. Sociodemographic profile of participants

Tables A1–A3 show the sex, age and employment status of FGD participants and Table A4 the job positions of the key informants who participated in interviews.

**Table A1.** Sex of FGD participants

| **Sex** | **Target group** | | | **Total** |
| --- | --- | --- | --- | --- |
|  | **Patients/carers (non-IDPs)** | **IDPs** | **Health workers** |  |
| Men | 24 | 7 | 9 | 40 |
| Women | 40 | 18 | 16 | 74 |
| Total | 64 | 25 | 25 | 114 |

**Table A2.** Age of FGD participants

| **Age (years)** | **Target group** | | | **Total** |
| --- | --- | --- | --- | --- |
|  | **Patients/carers (non-IDPs)** | **IDPs** | **Health workers** |  |
| 18–24 | 10 | 2 | 2 | 14 |
| 25–34 | 6 | 2 | 7 | 15 |
| 35–44 | 18 | 12 | 3 | 33 |
| 45–54 | 17 | 5 | 9 | 31 |
| ≥ 55 | 13 | 4 | 4 | 21 |
| Average | 42.6 | 41.3 | 42.7 | 42.3 |

**Table A3.** Employment status of FGD participants

| **Employment status** | **Target group** | | **Total** | |
| --- | --- | --- | --- | --- |
|  | **Patients/carers (non-IDP)** | **IDPs** |  |  |
| Working (has a job) | 36 | 10 | 46 | |
| Not working, unemployed | 9 | 3 | 12 | |
| Pensioner | 8 | 1 | 9 | |
| Homemaker | 2 | 0 | 2 | |
| On leave (parental or personal) | 3 | 4 | 7 | |
| Unable to work, including due to disability | 4 | 4 | 8 | |
| Student, pupil | 2 | 2 | 4 | |
| Stay-at-home mother | 0 | 1 | 1 | |
| Total | 64 | 25 | 89 | |
| Health workers | | | | |
| Health professional | | | | 12 |
| Health management and support personnel | | | | 8 |
| Local CSO representative | | | | 5 |
| Total | | | | 25 |

**Table A4.** Job position of key informants

| **Position** | **Quantity** |
| --- | --- |
| Subnational/local government official | 16 |
| Facility manager | 10 |
| Senior clinician | 8 |
| Total | 34 |

## Part B. Interview guide and FGD guides

### 1. Guiding script and topic guide - FGDs with patients resident in localities within ~50 km of active hostilities

**INTRODUCTION**

Hello! My name is ________. I am the representative of UISR, which, in partnership with the World Health Organization, is carrying out this research project, which focuses on community perspectives on health system recovery in Ukraine. As already mentioned, we’re interested in your experiences of accessing care during the war - what barriers you’ve faced, what has helped you still get care when you needed it, and what you think should be prioritised for recovery now and for rebuilding over the longer term.

As a reminder, today’s group includes residents living within ~50 km of active hostilities who have sought care for themselves or a household member within the last six months. First let me define what we mean by this term - “health system recovery”. What we mean by this is the things that are being done to restore and strengthen the health care system in the parts of the country most affected by the war. These things might be done by, for instance, central, regional or local government authorities, or by either Ukrainian or international/foreign humanitarian organisations, or charities, or community groups.

Recovery includes things such as:

- the rebuilding and/or repair of health care facilities;
- the restoration and/or replacement of medical equipment;
- alternative (and short-term) forms of health care provision – e.g., mobile clinics
- things that are being done to support health professionals to return to work;
- things that are being done to improve your access to care – for example, to see a family doctor and get hold of the medicines you need.

Participation in this discussion is voluntary. If you feel it is right to refrain from answering any question, simply say that you do not want to answer that question. This is absolutely fine; we will just give the floor to another participant. You may stop participating in the discussion at any time, and you do not have to answer any question.

We will analyse and report the data without identifying individual participants or organisations. To make our discussions interesting and productive, I would like to ask you to be as sincere as possible when answering the questions. Please do not worry about whether your opinion matches the opinions of other participants. There are no right or wrong answers. We are not trying to evaluate your knowledge or convince you of anything! Nor will we condemn or approve of your views in any way. Say what you think you need to say.

You may speak the language that is comfortable for you, and to express yourself in the form that comes to your mind. With your permission, I will use mainly Ukrainian.

By the way, I have a big favour to ask. Please do not rush to express your opinion, interrupting others. However, in order to optimise the communication, I may have to intervene. Sometimes I will interrupt you – for which I apologise! That will be not because we are not interested in your opinion, but only because we have very limited time, and we have many different questions to address. If you are not expecting any urgent messages, I would kindly ask you to switch your mobile phones to silent mode. Or, even better, to turn them off.

Our discussion will be audio recorded. This is solely to ensure that we keep an accurate record of the discussion, so that can process the results of the discussion after our meeting. The members of the research team will process the information and write a report based on the opinions that you, and other participants in the discussion, share with us. Because this is a group discussion, we ask everyone to respect each other’s privacy and not repeat what others say outside the group. The recording will be stored securely, transcribed with identifying details removed, and the audio file will be deleted once transcription is complete.

According to the plan, the duration of our discussion is about 1.5-2 hours. You have already received information about the study; before we start recording, can I confirm that you consent to take part and to the discussion being audio recorded?

Then let’s proceed.

**Let’s start with introducing ourselves. Please tell us how we can address you during the discussion.**

*ASK EACH RESPONDENT TO DECIDE HOW THEY CAN BE ADDRESSED DURING THE DISCUSSION*

Thank you!

Now, let’s get into the questions.

1. How has the close proximity of the war affected your access to health care in the local area (including services and medicines)? *[Prompts: we are interested in their experiences as people located close to active conflict areas, in which local health facilities are being damaged; equipment destroyed, health workers and civilians have left etc, - how is this impacting on them in terms of their access to the health care they need?]*

2. How well do you think the local health system has responded to these effects? What has gone well? What have you been impressed or surprised by (if anything)? What could have been better (if anything)? *[Follow-up questions: Since February 2022, what has helped to ensure you can access care when you need it (for example, changes in how services are organised, outreach/mobile services, teleconsultations, referral arrangements, support with transport)? In your view, what have health facilities, local authorities, or humanitarian organisations done that has been most effective in helping people access care/ keeping services running? Were there any changes introduced during the war that did not work well or created new barriers?]*

3. What services have been most badly disrupted, in your experience – e.g., local clinics; hospitals; pharmacies? Are there new, or bigger, barriers to access due to the war (e.g., have administrative barriers increased; or cost barriers; other barriers?)

4. Are you able to access care in the same way, from the same providers, as before the war? Or have you changed the way you access care? *[Prompts: For example, are you having to go further afield to get the care or medicines you need; or can you access alternative forms of provision (e.g., mobile clinics/pharmacies)? What are the related costs, difficulties or inconveniences for patients in this context?]*

5. Are you satisfied with what has been done to sustain access to health care locally? What has gone well? What could have been better?

6. Have you heard about the Program of Medical Guarantees (PMG) or reform of the primary healthcare level? To what extent do you think this has affected (positively or negatively) your ability to access care despite the effects of the war?

7. Do you know about the “Affordable Medicines Program”? To what extent do you think this has affected (positively or negatively) your ability to access medicines despite the effects of the war?

8. Do you see evidence of health sector recovery in your local community?

*[Prompts: remind them of what we mean by recovery: the rebuilding and/or repair of health care facilities; the restoration and/or replacement of medical equipment; things that are being done to support health professionals to return to work; alternative forms of health care provision – e.g., mobile clinics, etc. - and the actions of humanitarian agencies.]*

9. Which, if any, of these recovery activities have been important to you in terms of your ability to access the health services and medicines you need?

10. What changes would you like to see in your local community in the near future (e.g., the next year)? I.e., what changes do you think would make the greatest impact on: the availability of, or your level of access to, the health services and medicines you need?

11. What kinds of (health-related) recovery activities will be most impactful in enabling people who’ve left your community due to the proximity of the war to return to it when the security situation permits?

12. How important do you think the condition of the health system is in your / other people’s thinking about the possibility of making such a return (again, once the security situation permits)?

***THIS IS THE CONCLUSION OF OUR DISCUSSION. THANK YOU! IT WAS VERY INTERESTING AND INFORMATIVE.***

### 2. Guiding script and topic guide - FGDs with health workers in facilities within ~50 km of active hostilities / local stakeholders

**INTRODUCTION**

Hello! My name is _____________. I am the representative of UISR, which, in partnership with the World Health Organization, is carrying out this research project, which focuses on community perspectives on health system responses and recovery in Ukraine.

We want to understand how the war has affected service delivery and access in your area, what adaptations have helped you sustain essential services despite these challenges, and what priorities you see for recovery now and reconstruction over the longer term.

So that we’re all on the same page, let me define what we mean by this term - “health system recovery”.

Health system recovery refers to the things that are being done to restore and strengthen the health system in response to the damage and disruption caused by war – especially in the parts of the country and population groups most affected by the war.

This includes things such as:

- the rebuilding and/or repair of health care facilities;
- the restoration and/or replacement of medical equipment;
- alternative (and short-term) forms of health care provision – e.g., mobile clinics;
- things that are being done to support health professionals to return to work; and
- things that are being done to improve patient’s access to health care – for example, to see a family doctor and get hold of the medicines they need.

We are interested in recovery efforts undertaken by central government, and international/foreign humanitarian organisations – but also by organisations such as your own.

So, we’re interested in your experiences of health system recovery – including those you’ve led, or engaged on. And we’d also like to talk about:

- your perceptions of recovery efforts to date – their successes and failures;
- the importance of health system recovery for the future sustainability of communities;
- changes you’d like to see, both in the near future – and in the longer-term; and
- opportunities for, and barriers to, the changes you’d like to see.

Participation in the discussion is voluntary. If you feel it is right to refrain from answering any question, simply say that you do not want to answer that question. This is alright; we will just give the floor to another participant. We will analyse and report the data without identifying individual participants or organisations.

You may stop participating in the discussion at any time, and you do not have to answer any question.

To make our communication interesting and productive, I would like to ask you to be as sincere as possible when answering the questions suggested. Please do not worry about whether your opinion matches the opinions of other participants of the discussion and to what extent. Say what you think you need to say.

We would like to note that there are no right or wrong answers. We intend neither to evaluate your knowledge or to convince you of anything, nor condemn or approve you in any way. You may speak the language that is comfortable for you, and to express yourself in the form that comes to your mind. With your permission, I will use mainly Ukrainian.

By the way, I have a big favour to ask. Please do not rush to express your opinion, interrupting others. However, in order to optimise the communication, I may have to “wedge” into someone’s monologue or dialogue. I would like to apologize in advance to some of the participants: sometimes I will interrupt you. That will be not because we are not interested in your opinion, but only because we have very limited time and have to discuss many different topics.

If you are not expecting any urgent messages, I kindly ask you to switch your mobile phones to silent mode. Or, even better, to turn them off. Our discussion will be audio recorded. This is solely to ensure that we keep an accurate record of the discussion, so that can process the results of the discussion after our meeting. The members of the research team will process the information and write a report based on the opinions that you, and other participants in the discussion, share with us.

Because this is a group discussion, we ask everyone to respect each other’s privacy and not repeat what others say outside the group. The recording will be stored securely, transcribed with identifying details removed, and the audio file will be deleted once transcription is complete.

According to the plan, the duration of our discussion is about 1.5-2 hours. You have already received information about the study; before we start recording, can I confirm that you consent to take part and to the discussion being audio recorded

Then let’s proceed.

**Let’s start with introducing ourselves. I ask everyone present to briefly tell about themselves. I will give you an example:**

My name is ___________.

ASK EACH RESPONDENT TO INTRODUCE THEMSELVES (WORK EXPERIENCE IN HEALTHCARE WITHOUT DEEP DETAILS, ANY OTHER INFORMATION THEY CONSIDER NECESSARY.)

1. How has the war affected the health system in your local area? What have been its effects on the availability of health services and medicines for the local population? Are different parts of the health system (e.g., primary care, specialist care, inpatient care, pharmacies) affected in different ways/to different extents? *[Prompt: it may be worth emphasising to the participants that we are interested in impacts related to the close proximity of active conflict – so, we are looking to understand the distinctive impacts of the war for the most affected communities, rather than the war’s impacts more generally.]*

2. How well do you think the local health system has responded to disruptions caused by the close proximity of the war’s front-line – for instance, direct damage to facilities and equipment, outward migration of communities and health workers, etc? What do you think has gone well? What could have gone better?

3. Are patients able to access health care in the same way as before the war? Or are they, for example, having to go further afield? Or accessing care in new ways (e.g., in mobile clinics/pharmacies)? If there are changes, how are these affecting their levels of access to quality care?

4. What, if any, difference do you think the Program of Medical Guarantees (PMG) has made on patients’ access to health services in the context of the war? How – and in what ways?

5. And can I ask the same question about the “Affordable Medicines Program”?

6. What specific adaptations have you observed and/or implemented in your facility/by your team to sustain essential services (e.g., triage, task-shifting, altered clinic hours, service consolidation, outreach/mobile, telemedicine, referral redesign)?

7. What factors made these adaptations possible (or limited them) - for example, financing arrangements, staffing, leadership, partner support, security conditions?

8. Which adaptations introduced during the war should be retained as part of longer-term recovery/reconstruction, and which should be reversed? What might be expanded or scaled-up?

9. What health care-related recovery efforts do you see in your local area? Can you give examples? Which have been the most important in terms of impacts on patients’ access to care? *[Prompts: remind them of what we mean by recovery: the rebuilding and/or repair of health care facilities; the restoration and/or replacement of medical equipment; things that are being done to support health professionals to return to work; alternative forms of health care provision – e.g., mobile clinics, etc. - and the actions of humanitarian agencies.]*

10. What kinds of (health-related) recovery activities would you like to see – i.e., which would be most impactful in terms of patients’ access to care, and enabling people who’ve left the community (due to the proximity of the war) to return to it when the security situation permits?

11. What kinds of support (e.g., from government, other Ukrainian actors, or international partners) will be needed to enable those activities to happen?

12. How important do you think the condition of the health system is in your / other people’s thinking about the possibility of making such a return (again, once the security situation permits)?

13. Are there any additional points you would like to raise – and that we can bring to the attention of the national and international policymakers that will read our report?

**THIS IS THE CONCLUSION OF OUR DISCUSSION. THANK YOU! IT WAS VERY INTERESTING AND INFORMATIVE.**

### 3. Guiding script and topic guide - FGDs with internally displaced persons

**INTRODUCTION**

Hello! My name is _______________. I am the representative of UISR, which, in partnership with the World Health Organization, is carrying out this research project, which focuses on community perspectives on recovery of the health system in Ukraine.

We’d like to hear about your experiences of seeking care in your host community - any barriers you’ve faced, what has helped you access services, and what you think should be prioritised to support recovery now and rebuilding in the longer term.

So, we’ll be asking questions about your experiences of (for example):

- accessing health care as a person displaced from your home community after 24 February 2022 due to the full-scale invasion;
- any barriers (financial, administrative, logistical, other) that you face or have faced in accessing health care;
- the degree to which you are exposed or have been exposed to health care-related expenditures;
- your experience with health system recovery in your *host* community (i.e., the place in which you have moved to, due to the full-scale invasion);
- your expectations of, and preferences for, recovery in your host and *home* communities; and
- how important health system recovery in your home community is to you - in terms of your ability to return to that community (should you wish to do so).

It might be helpful if I define this term “recovery”, as it applies to the health care system.

By recovery, I’m referring to the things that are being done to restore and strengthen the health care system in response to the war’s impacts.

These things might be undertaken by, for instance, central, regional or local government authorities, Ukrainian or international/foreign humanitarian organisations, charities, community groups, and so on.

They include things like:

- the rebuilding and/or repair of health care facilities;
- the restoration and/or replacement of medical equipment;
- the introduction of alternative forms of health care provision – for example, mobile clinics;
- things that are being done to support health professionals to return to work;
- the introduction of new financial or administrative arrangements designed to improve patients’ access to health care – for example, to see a family doctor and get hold of the medicines they need.

Participation in this discussion is voluntary.

If you feel it is right to refrain from answering any question, simply say that you do not want to answer that question. This is absolutely fine; we will just give the floor to another participant. You may stop participating in the discussion at any time, and you do not have to answer any question.

We will analyse and report the data without identifying individual participants or organisations.

To make our communication interesting and productive, I would like to ask you to be as sincere as possible when answering the questions. Please do not worry about whether your opinion matches that of other participants. There are no right or wrong answers. We are not trying to evaluate your knowledge or convince you of anything!

Nor will we condemn or approve of your views in any way. Please - say what you think you need to say.

You may speak the language that is most comfortable for you, and express yourself in the form that comes to your mind. With your permission, I will mainly use Ukrainian.

By the way, I have a big favour to ask: please do not rush to express your opinion, interrupting others. However, in order to optimise the communication, I may have to intervene. Sometimes I may even need to interrupt you – for which I apologise in advance! This will not be because I am not interested in your opinion, but only because we have limited time, and many different questions to address.

If you are not expecting any urgent messages, I would kindly ask you to switch your mobile phones to silent mode. Or, even better, to turn them off.

Our discussion will be audio recorded. This is solely to ensure that we keep an accurate record of the discussion, so that can process the results of the discussion after our meeting.

The members of the research team will process the information and write a report based on the opinions that you, and others participants in the discussion, share with us. Because this is a group discussion, we ask everyone to respect each other’s privacy and not repeat what others say outside the group. The recording will be stored securely, transcribed with identifying details removed, and the audio file will be deleted once transcription is complete.

According to the plan, the duration of our discussion is about 1.5-2 hours.

You have already received information about the study; before we start recording, can I confirm that you consent to take part and to the discussion being audio recorded

Then let’s proceed.

**Let’s start with introducing ourselves. Please tell us how we can address you during the discussion.**

*ASK EACH RESPONDENT TO DECIDE HOW THEY CAN BE ADDRESSED DURING THE DISCUSSION*

Thank you!

Now, let’s get into the questions.

1. What has been your experience of seeking health care as a displaced person? For example, do you think your status as a displaced person has affected (positively or negatively) your ability to see a family doctor in your host community? Or access specialist or hospital care, or medicines?

2. For those of you who have experienced negative effects, what has been the cause?

*[Prompts: for instance, have you had to pay, or pay more, for services that in your host community would have been free, or would have cost less? Or are there administrative barriers (e.g., difficulties in making a new registration with a local doctor, or difficulties in being referred to a specialist or hospital?]*

3. What, if anything, did health facilities or local authorities in your host area do to adapt services for displaced people (e.g., registration support, information provision, additional clinics, outreach/mobile services)?

4. What sources of information or assistance helped you most to understand entitlements and navigate care (e.g., family doctor registration, hotlines, NGOs, social workers), and what was missing?

5. Have you had experience with being denied care due to your status as a displaced person? Or, conversely, have you found that your levels of access have improved due to your status – relative to your previous experience or the experience of other, non-displaced, persons?

6. Do you feel that displaced people and long-standing residents are treated equally when seeking care? What shapes that experience?

7. We’re aware that many IDPs choose to retain their registration (their “declaration”) with their “old” family doctor – rather than registering with a new doctor in their new location. Why might this be? *[Prompts: does this process involve too much paperwork? Or is it more that there’s a lack of information about how to do it? Or is due to the unavailability of doctors? Or some combination of these factors? Which of these factors do you think is the most important?]*

8. Do you think that registration with a local doctor makes a difference to your levels of access to health care? E.g., is it possible to access free medicines and/or referrals to specialist care without registration with a local doctor? If you don’t think it makes much difference, can you tell us why?

9. Have you heard about the Program of Medical Guarantees *(moderator should provide clarifying information on what this is)*? What, if any, difference do you think the PMG/AMP has made to your ability to access the services you need in the context of your displacement? How? *[Prompt. E.g., what, if any, difference has the AMP made on the cost of services or medicines you utilise?]*

10. What about the “Affordable Medicines Program” *(moderator should provide clarifying information on what this is)*? Do you think this has affected your ability to obtain the medicines you need in the context of your displacement? How?

11. Do you see evidence of health sector recovery in your host community?

*[Prompts: remind them of what we mean by recovery: the rebuilding and/or repair of health care facilities; the restoration and/or replacement of medical equipment; things that are being done to support health professionals to return to work; alternative forms of health care provision – e.g., mobile clinics, etc. - and the actions of humanitarian agencies.]*

12. Which, if any, of these examples of recovery has been important to you in terms of your ability to access the health services and medicines you need?

13. What changes would you like to see in your host community in the near future (e.g., the next year)? I.e., what changes do you think would make the greatest impact on the availability of, or your level of access to, the health services and medicines you need?

14. What are your expectations about the future of the local health system in your home community (i.e., the community you have been displaced from)? If the security situation in your home community was to improve sufficiently, what would you expect the situation in the local health system would be – i.e., would it be possible for you to get access to the care you need? What kinds of recovery efforts will be needed for people to return to that community?

15. How important do you think the condition of the health system is in your thinking about the possibility of return (again, once the security situation permits)?

We will conclude at this point.

***THIS IS THE CONCLUSION OF OUR DISCUSSION. THANK YOU! IT WAS VERY INTERESTING AND INFORMATIVE.***

### 4. Guiding script and topic guide for key informant interviews

**INTRODUCTION**

Good morning/afternoon Mr/Mrs________________________(Surname). Thank you for taking the time to meet with our team today, we really appreciate it. We will shortly begin with a round of introductions, and then we will explain the purpose of our research and what you can expect from the interview.

As outlined in the information sheet and informed consent form, we are trying to understand your perspective on health system recovery.

Specifically, we’re exploring how war-related shocks have affected health system functioning and access, what adaptations have been used to sustain essential services, and what priorities you see for near-term recovery and longer-term reconstruction.

We are interviewing you today because you have direct involvement in this area, and we believe we can learn a lot from your knowledge, insights and expertise.

Today’s interview will not take more than 60-90 minutes. We would prefer if it could be as open as possible. You are the expert, and we would like to learn as much as possible from you. The interview will be treated as confidential within the research team – and your name and organization will not be identified in any final outputs from the research.

We would like to now ask for your permission to audio-record our conversation. As the information sheet makes clear, the recording will be stored on a password-protected file on a WHO computer. It will be transcribed, and the information used in the course of the research. The recording itself will be destroyed once transcription has been completed. The transcription will not include any identifying information about you – nonetheless, it will also be stored on a password-protected file on a WHO computer.

Before we begin, I’d like to confirm consent. You have received the participant information sheet; do you have any questions? If you are participating in person, we will ask you to sign the consent form. If the interview is online, we will record your oral consent before we start.

Otherwise, do you have any questions with regards to today’s interview before we press record, and proceed with our questions?

**Questions:**

We’re going to ask some questions about health system responses and recovery. By the latter term, just to ensure we’re all clear, we mean activities that are intended to restore and strengthen the health care system in response to the damage and disruption caused by the current war – especially in the parts of the country, and/or among the population groups, that are most severely affected by the war.

1. How has the war affected the availability of health services and medicines for local people in this area? What are the impacts of the proximity of the war on the availability of health services and medicines?

2. *[If changes to availability are observed].* What are the main causes of these changes? [Prompts: are the main causes things like direct damage to facilities/pharmacies; or outward migration (of communities and/or health workers); or economic effects? Other causes?]

3. Which essential services have been most difficult to sustain, and what adaptations have been used to keep them running?

4. Do the war-related disruptions affect patients’ ability to access some types of care more than others – e.g., access to local family doctors, polyclinics, or hospitals; or to medicines? What causes these differences?

5. Are people in your region/area/your patients able to access health care in the same way as they did before the war? Are they having to go further afield to access care? Are they accessing care in new ways (e.g., in mobile clinics/pharmacies/outreach services)? If there are changes, how are these affecting their levels of access to quality care?

6. How well has the local health system responded to the challenges associated with its proximity to the war, in your opinion? What has gone well/less well?

7. How were key adaptation decisions made (facility/oblast/national), and how well were humanitarian actors integrated into these arrangements?

8. What data or community feedback mechanisms (if any) were used to identify access barriers and adjust services?

9. How can the local health system be better supported to respond to the challenges? What are the main priorities for support? Who needs to do what?

10. What health system recovery activities have happened/are happening in your local area? Can you give examples – including any that you’ve been directly involved in, or have supported, or have been affected by? *[Prompt: interviewer can also give some examples here, if needed – additional rebuilding and/or repair of health care facilities; restoration and/or replacement of medical equipment; provision of modalities for of health care provision – e.g., mobile clinics; additional financial support to encourage health professionals to return to work in these areas; additional financial/other forms of support for patients, to improve their access to health care.]*

11. Of these, which (if any) do you think has been important in helping you, or other local health system leaders, to maintain or improve local people’s access to care?

12. What, if any, role do you think the PMG has played in shaping patients’ access to services in your area during the war? How – and in what specific ways?

13. And can I ask the same question about the ‘Affordable Medicines Program’ - what, if any, role has it played in access to medicines in your area during the war? How, and in what ways?

14. What kinds of (health-related) recovery activities would you like to see – i.e., which do you think would be most impactful in terms of patients’ access to care? What kinds of support (e.g., from government, other Ukrainian actors, or international partners) do you think will be needed for those activities to happen?

15. How important do you think the state of the local health system is for people’s ability to return? *[Prompt: when the security situation allows, we want people to return. Is that in your thoughts now as you think about recovery in the health sector? Do you think people actively consider healthcare-related issues when thinking about returning – and what can you do to improve the health system so that more people think a return is going to be viable for them and their families.]*

16. Are there any additional points you would like to raise – and that we can bring to the attention of the national and international policymakers that will read our report?

**Thank you very much for your time and the information you shared with us!**
